# Nickel Nanoparticles Exposure and Reproductive Toxicity in Healthy Adult Rats

**DOI:** 10.3390/ijms151121253

**Published:** 2014-11-17

**Authors:** Lu Kong, Meng Tang, Ting Zhang, Dayong Wang, Ke Hu, Weiqi Lu, Chao Wei, Geyu Liang, Yuepu Pu

**Affiliations:** 1 Key Laboratory of Environmental Medicine Engineering, Ministry of Education, School of Public Health, Southeast University, Nanjing 210009, China; E-Mails: konglu@seu.edu.cn (L.K.); zhangting1207@gmail.com (T.Z.); 18051969217@163.com (K.H.); starvich@126.com (W.L.); seuweichao@163.com (C.W.); gyliang@seu.edu.cn (G.L.); 2 Key Laboratory of Developmental Genes and Human Disease in Ministry of Education, Medical School of Southeast University, Nanjing 210009, China; E-Mail: dayongw@seu.edu.cn

**Keywords:** nickel nanoparticle, reproductive toxicity, one-generation, rats

## Abstract

Nickel is associated with reproductive toxicity. However, the reproductive toxicity of nickel nanoparticles (Ni NPs) is unclear. Our goal was to determine the association between nickel nanoparticle exposure and reproductive toxicity. According to the one-generation reproductive toxicity standard, rats were exposed to nickel nanoparticles by gavage and we selected indicators including sex hormone levels, sperm motility, histopathology, and reproductive outcome *etc*. Experimental results showed nickel nanoparticles increased follicle stimulating hormone (FSH) and luteinizing hormone (LH), and lowered etradiol (E_2_) serum levels at a dose of 15 and 45 mg/kg in female rats. Ovarian lymphocytosis, vascular dilatation and congestion, inflammatory cell infiltration, and increase in apoptotic cells were found in ovary tissues in exposure groups. For male rats, the weights decreased gradually, the ratio of epididymis weight over body weight increased, the motility of rat sperm changed, and the levels of FSH and testosterone (T) diminished. Pathological results showed the shedding of epithelial cells of raw seminiferous tubule, disordered arrangement of cells in the tube, and the appearance of cell apoptosis and death in the exposure group. At the same time, Ni NPs resulted in a change of the reproductive index and the offspring development of rats. Further research is needed to elucidate exposure to human populations and mechanism of actions.

## 1. Introduction

Nickel is a silver-white metallic chemical element that is naturally present in the Earth’s crust [1]. Because of its unique physical and chemical properties, being tough, harder than iron, ferromagnetic, having good plasticity and highly resistant to rusting and corrosion, nickel and its compounds are widely used in industry [2]. Nickel is an essential element for at least several animal species. These animal studies associate nickel deprivation with depressed growth, reduced reproductive rates, and alterations of serum lipids and glucose [3].

Nickel is known as a potentially harmful element for humans. Its concentration in the environment can rise due to industrial activities [4,5,6,7,8,9,10]. Human exposure to nickel or its compounds has the potential to produce a variety of pathological effects, which may include cutaneous inflammations such as swelling, reddening, eczema and itching on skins, and may also include allergy reactions and teratogenicity in the human body. The most important adverse health effects due to nickel exposure are lung fibrosis and lung cancer [10,11]. Epidemiological studies have indicated that occupational exposure to nickel increased the incidence of some human cancers, such as lung, head, neck and nasal cancers, and so forth [12,13,14,15,16,17,18,19,20,21,22,23,24,25,26]. Nickel compounds have long been classified as human carcinogens according to the International Agency for Research on Cancer [27,28]. Furthermore, excessive nickel micro-particles (Ni MPs) can induce reproductive toxicity. Nickel ions exert a wide variety of adverse effects on reproduction and development, including influence on male and female subfertility or fertility, abortions, malformations and birth defects [29,30,31]. For example, Ni MPs treatment can decrease the reproductive capacity of zebra-fish and the reproduction success of the *S. litura* Noctuid moth [4,32]. Soluble nickel salts have been demonstrated to disturb mammalians and model organism reproductive functions [33,34]. Hormonal effects may play an important role in the reproductive toxicology of nickel both at the neuroendocrine and gonadal levels in the hypothalamic–pituitary–gonadal (HPG) axis [33].

With the rapid development of nanotechnology, the application of nanomaterials is becoming more and more extensive. Manufactured nanomaterials, defined as materials with at least one dimension ranging from 1 to 100 nm, possess unique or even increased physicochemical properties, such as nanoscale size effects, quantum effects, expanded surface area as well as unique electric, thermal, mechanical, and imaging properties. These special characteristics show promise for nanomaterials to be used in a wide range of applications [35]. At present, nanomaterials are beginning to influence human life in many ways, therefore understanding the environmental health and safety aspect of nanoparticles has become a crucial problem [36]. Metallic nanoparticles including metallic nickel nanoparticles (Ni NPs) are among the most widely used types of nanomaterials [37]. Ni NPs give a product with many new characteristics, including a high level of surface energy, high magnetism, low melting point, high surface area, and low burning point. Ni NPs are used in many fields, such as for catalysts, for magnetic materials, in biological medicine and for conductive paste *etc.* So human and environmental exposures to Ni NPs become inevitable, but health and environmental impacts have not been fully investigated. According to some studies, Ni NPs may induce liver and spleen injury, lung inflammation, cardiac toxicity [38], and exhibit higher carcinogenic potential than fine particles [39]. As mentioned before, Ni MPs have been shown to have reproductive toxicity, and Ni NPs may also induce reproductive toxicity [40]. However, there is still a gap in the evaluation of their reproductive toxicity. The goal of the present study was to evaluate the reproductive toxicity of Ni NPs to rats.

## 2. Results

### 2.1. Characterization of Ni NPs

**Figure 1:**
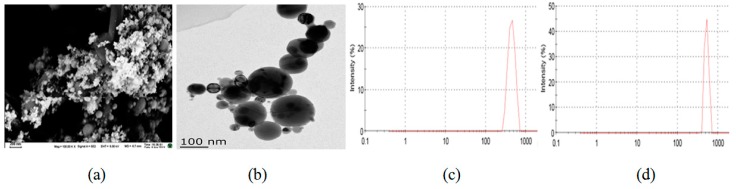
SEM (scale bar = 200 nm) (**a**) and TEM (scale bar = 100 nm) (**b**) images and particle size distributions of nickel nanoparticles (Ni NPs) of 5 μg/mL (**c**) and 12.5 μg/mL (**d**).

The SEM and TEM images showed that Ni NPs were spherical in shape. The size distribution varied from 30 to 100 nm in diameter, the average size of Ni NPs was 90 nm, and there was slight agglomeration. In the dispersion, the particle size of Ni NPs of 5 μg/mL had a distribution from 260 to 725 nm, and peak size was about 444 nm. However, the average particle size of 12.5 μg/mL had a distribution from 400 to 879 nm, and peak size of about 522 nm. See Figure 1. Meanwhile the average size distribution of Ni MPs is 3.34 ± 0.67 µm without agglomeration.

### 2.2. Body Weights and Organ Weight Coefficients

No female or male deaths occurred during the study and clinical observations did not show any significant findings. Data for female and male rat body weight gain and organ weight coefficients appear in Table 1. No statistically significant difference was found in female body weights and coefficients of liver, kidney, and ovary between the control and Ni NPs-exposed groups. However the mean body weight of Ni MPs at week 13 decreased significantly from controls. The ratio of lung weight over body weight of female rats increased gradually on increasing the dose of Ni NPs. Furthermore, lung weight coefficients for female rats treated with Ni MPs increased significantly compared to control and the high dose of Ni NPs. For male rats, initial weights of different groups had no difference significantly. The differences in body weight at the first week to the final week of the experimental treatments were large, and compared with the control group, the body weights of the high dose group and Ni MPs increased significantly. At the same time, the experiments showed organ weight coefficients of lung, testis, and epididymis increased significantly when treated with high dose Ni NPs and Ni MPs compared with the control. Moreover, lung coefficients for male rats treated with Ni MPs increased significantly compared with the high dose of Ni NPs.

**Table 1:** Mean body weight and organ weight coefficients for female and male rats.

Group	Control	Low Dose	Mid Dose	High Dose	Ni MPs
Female parental rats					
Mean body weight (g)					
zero week	91 ± 6	93 ± 7.20	93 ± 7	93 ± 7	92 ± 6
first week	160 ± 16	165 ± 17	166 ± 8	161 ± 20	160 ± 15
fourth week	235 ± 17	234 ± 24	233 ± 15	231 ± 26	229 ± 19
seventh week	269 ± 19	276 ± 26	277 ± 19	271 ± 25	267 ± 22
tenth week	329 ± 26	333 ± 34	336 ± 22	324 ± 28	313 ± 29
thirteenth week	355 ± 43	359 ± 49	340 ± 23	337 ± 33	326 ± 30 *
sixteenth week	322 ± 31	337 ± 40	325 ± 20	322 ± 34	316 ± 28
final weight	307 ± 28	313 ± 29	314 ± 21	307 ± 28	300 ± 24
Organ weight coefficient (%)					
liver	2.84 ± 0.55	2.94 ± 0.52	2.65 ± 0.32	2.90 ± 0.49	2.76 ± 0.41
kidney	0.65 ± 0.06	0.65 ± 0.06	0.63 ± 0.05	0.65 ± 0.05	0.64 ± 0.06
lung	0.50 ± 0.07	0.51 ± 0.08	0.53 ± 0.08	0.53 ± 0.09 ^1^	0.61 ± 0.13 *
ovary	0.05 ± 0.01	0.05 ± 0.01	0.05 ± 0.01	0.05 ± 0.01	0.05 ± 0.01
Male parental rats					
Mean body weight (g)					
zero week	100 ± 7	98 ± 9	99 ± 7	99 ± 10	100 ± 8
first week	196 ± 12	196 ± 13	195 ± 13	185 ± 18	182 ± 11 *
third week	303 ± 14	301 ± 17	293 ± 21	284 ± 13 *	284 ± 13 *
fifth week	370 ± 13	376 ± 22	365 ± 23	357 ± 16	345 ± 25 *
seventh week	421 ± 18	419 ± 17	408 ± 23	401 ± 17 *	388 ± 21 *
ninth week	453 ± 20	459 ± 24	453 ± 23	434 ± 19 *	432 ± 23 *
eleventh week	487 ± 22	486 ± 22	477 ± 20	460 ± 18 *	457 ± 28 *
Organ weight coefficient (%)					
liver	2.13 ± 0.12	2.13 ± 0.12	2.14 ± 0.14	2.16 ± 0.13	2.16 ± 0.15
kidney	0.60 ± 0.05	0.60 ± 0.04	0.61 ± 0.03	0.60 ± 0.03	0.61 ± 0.04
lung	0.35 ± 0.04	0.37 ± 0.05	0.41 ± 0.05 *	0.43 ± 0.07 ^1^	0.57 ± 0.12 *
testis	0.61 ± 0.04	0.63 ± 0.05	0.65 ± 0.04	0.64 ± 0.03 *	0.67 ± 0.06 *
epididymis	0.19 ± 0.06	0.23 ± 0.01	0.23 ± 0.02 *	0.25 ± 0.02 *	0.23 ± 0.03

* *p* < 0.05, compared with control group (0 mg/kg body weight (BW)); ^1^ *p* < 0.05, compared with Ni MPs (45 mg/kg BW).

### 2.3. Sperm Motility of Parental Males

After 15 and 45 mg/kg/day Ni NPs exposures, linearity (LIN) decreased significantly, and curvilinear velocity (VCL) of the high dose group decreased significantly compared with control. However, the motility test of rat sperm showed Ni NPs induced the increase of beat cross frequency (BCF). At the same time, Ni MPs exposure decreased the motility of the parameters LIN and VCL, and increased BCF significantly. See Table 2.

**Table 2:** Effects of nickel nanoparticles (Ni NPs) on rat sperm motility.

Group	Control	Low Dose	Mid Dose	High Dose	Ni MPs
average path velocity (VAP) (μm/s)	210 ± 15	211 ± 14	209 ± 10	207 ± 8	204 ± 8
curvilinear velocity (VCL) (μm/s)	410 ± 24	405 ± 25	398 ± 18	382 ± 21 *	384 ± 29 *
straight line velocity (VSL) (μm/s)	145 ± 9	144 ± 9	144 ± 6	141 ± 6	140 ± 8
beat cross frequency (BCF) (Hz)	19 ± 1	20 ± 1 *	20 ± 1 *	20 ± 1 *	20 ± 1 *
straightness (STR) (%)	67 ± 1	68 ± 1	68 ± 1	67 ± 1	67 ± 1
linearity (LIN) (%)	37 ± 1	37 ± 1	36 ± 1 *	36 ± 1 *	36 ± 1 *
amplitude of lateral head displacement (ALH) (μm)	18 ± 1	18 ± 0	19 ± 1	19 ± 1	19 ± 1
elongation (ELON) (%)	68 ± 1	69 ± 1	68 ± 2	68 ± 1	68 ± 2

* *p* < 0.05, compared with control group.

### 2.4. Effect of Ni NPs on Serum Hormone Concentrations

**Figure 2:**
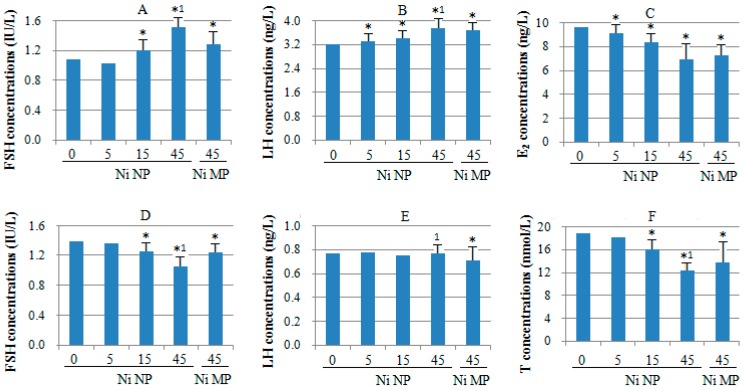
Effects of Ni NPs on serum hormone concentrations in rats. Follicle stimulating hormone (FSH) in females (**A**); Luteinizing hormone (LH) in females (**B**); Estradiol (E_2_) in females (**C**); FSH in males (**D**); LH in males (**E**); Testosterone (T) in males (**F**). Serum hormone concentrations were measured by ELISA. Values represent the mean ± SD (*n* = 7). * *p* < 0.05, compared with control group (0 mg/kg BW); ^1^ *p* < 0.05, compared with Ni MPs (45 mg/kg BW).

To determine whether Ni NPs exposure induces alterations to the female or male reproductive system, according to the one-generation reproductive toxicity study, we treated adult female Sprague-Dawley rats with Ni NPs at 5 mg/kg BW (low dose), 15 mg/kg BW (mid-dose), and 45 mg/kg BW (high dose) for eighteen weeks by gavage. Similarly we treated male rats with Ni NPs for ten weeks. Mid-dose and high dose of Ni NPs significantly increased serum FSH concentrations in female rats compared with controls (Figure 2A), and all doses of Ni NPs significantly increased LH in female rats (Figure 2B). However, high dose of Ni NPs significantly increased serum FSH and LH concentrations compared to Ni MPs (Figure 2A,B). In contrast, the serum E_2_ of the females was decreased by Ni NPs exposure (Figure 2C). Exposure to Ni NPs (mid-dose and high-dose) resulted in the same alteration of serum FSH and T concentrations in male rats as observed with Ni MPs (Figure 2D,F). Compared with Ni MPs, the levels of FSH and T in serum were significantly lower while the level of LH was significantly higher in the high dose of Ni NPs (Figure 2D–F).

### 2.5. Histopathology

**Figure 3:**
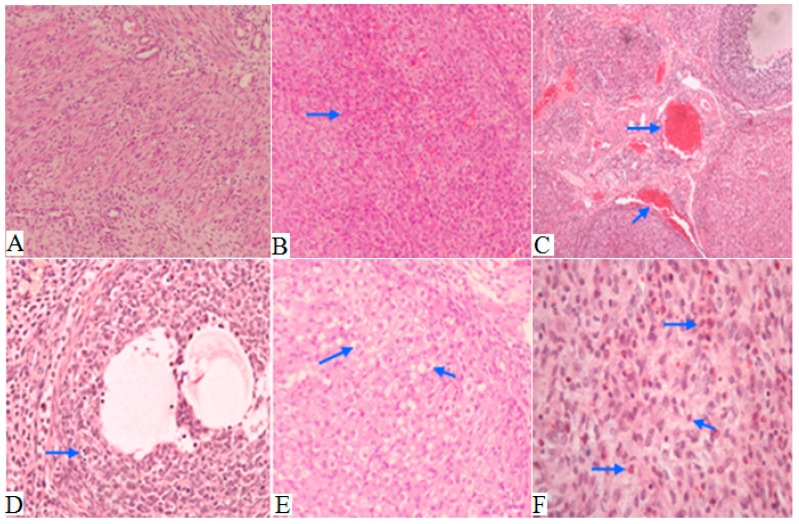
Effects of Ni NPs on histopathology of ovaries in female rats. The control group (**A**); 15 mg/kg BW (**B**); 45 mg/kg (**C**–**E**); Ni MPs group (**F**). Original magnification was 100×, 200× and 400× (100× refers to picture **A**, **B** and **C**; 200× refers to picture **D** and **E**; 400× refers to picture **F**). The arrow on (**B**) and (**C**) points to vascular dilatation and congestion, on (**D**) it points to Lymphocytes, on (**E**) it points to luteal cells, and on (**F**) it points to eosinophils and inflammatory cells.

**Figure 4:**
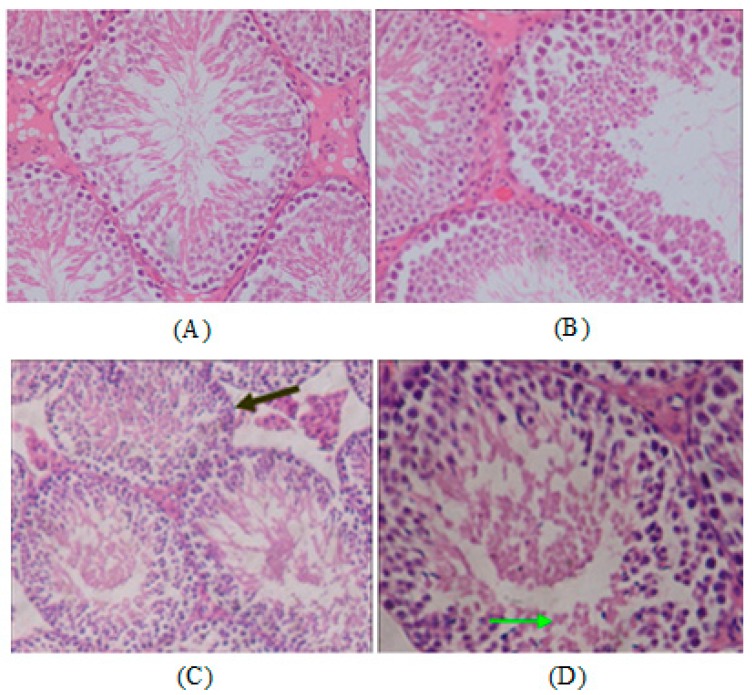
The testicular histopathological changes of the male rats. The control group (**A**); 45 mg/kg BW (**B**–**D**). Original magnification 200× and 400× (200× refers to picture **A**, **B** and **C**; 400× refers to picture **D**). The arrow on (**C**) points to disordered arrangement of cells in the tube and on (**D**) it points to cell apoptosis.

To confirm whether Ni NPs treatment may damage the reproductive system of rats, and change serum hormone levels (FSH, LH, E_2_ and T) caused by ovary and testis tissues damages, we examined the histopathology of ovaries and testes of parental rats. There were no adverse histopathological presentations observed in the control group (Figure 3A). However, pathological results showed vascular dilatation and congestion (Figure 3B,C), ovarian lymphocytosis (Figure 3D), luteal cells increasing and becoming cavitated (Figure 3E), increased eosinophils and inflammatory cell infiltration (Figure 3F) in rat ovaries tissue in the Ni NPs exposure group. At the same time, a histologic section of testis tissue showed seminiferous tubules lined by germ cells in various stages of development (the spermatogenic series), and containing luminal spermatozoa in the control group (Figure 4A), with the shedding of epithelial cells of the raw seminiferous tubule (Figure 4B), disorder arrangement of cells in the tube (Figure 4C), and appearance of cell apoptosis and death (Figure 4D) in the Ni NPs or Ni MPs group.

### 2.6. Reproductive Outcome

The effect of Ni NPs on reproductive index and offspring development of rats was studied. (See Table 3 and Table 4). The results demonstrated that birth survival rate in fetal rats to parents with Ni NPs and Ni MPs significantly decreased compared with fetal rats of parents without Ni NPs or Ni MPs (control), and the birth survival rate of the high dose of Ni NPs significantly decreased compared with Ni MPs. At the same time, the feeding survival rates in the Ni NPs and Ni MPs groups were also lower than the control group. However, the feeding survival rate of the high dose Ni NPs was higher than that of Ni MPs. Out of 104 live born rats 67 survived (64.4%) after 21 days at a high dose of Ni NPs and out of 174 live born rats 75 survived (43.1%) after weaning (21 days) in Ni MPs. Ni NPs could also reduce the chance of mating success and pregnancy in rats, but there was no statistical significance (*p* > 0.05). Ni NPs caused weight gain loss in the neonatal rats at 4, 7, 14, and 21 days of age (Table 4). The offspring weights of mid-dose and high dose of Ni NPs and Ni MPs significantly decreased compared with the control group. However, there was no statistical significance between high dose of Ni NPs and Ni MPs.

**Table 3:** Effects of Ni NPs on the rats reproductive index (%).

Group	Mating Success Rate	Pregnancy Rate	Live Birth Rate	Birth Survival Rate	Feeding Survival Rate
control	100 (20/20)	100 (20/20)	100 (20/20)	94 (185/196)	79 (147/185)
low dose	90(18/20)	90 (18/20)	100 (18/18)	86 (171/198) *	73 (125/171)
mid dose	80 (16/20)	80 (16/20)	100 (16/16)	75 (142/190) *	65 (93/142) *
high dose	80 (16/20)	80 (16/20)	100 (16/16)	67 (104/156) *^,1^	64 (67/104) *^,1^
Ni MPs	90 (18/20)	90 (18/20)	100 (18/18)	82 (174/211) *	43 (75/174) *

* *p* < 0.05, compared with control group; ^1^ *p* < 0.05, compared with Ni MPs (45 mg/kg BW).

**Table 4:** Body weight changes of pup rats during the experiment (g).

Group	Birthday	The 4th Day	The 7th Day	The 14th Day	The 21th Day
Control	7 ± 1	11 ± 2	16 ± 3	31 ± 3	50 ± 5
Low dose	7 ± 1	10 ± 2 *	15 ± 3	27 ± 5 *	48 ± 5
Mid dose	7 ± 0	10 ± 2 *	14 ± 3 *	25 ± 7 *	46 ± 6 *
High dose	7 ± 0	10 ± 2	14 ± 2	24 ± 2 *	42 ± 6 *
Ni MPs	7 ± 0	9 ± 2 *	13 ± 3 *	26 ± 2 *	45 ± 7 *

* *p* < 0.05, compared with control group.

## 3. Discussion

Nanomaterials, such as gold, silver, and gold-silver alloy nanoparticles were shown to have reproductive toxicity by impairing key sperm functions, somatic and reproductive cells, and mammalian gametes [41,42,43]. The results of the current study demonstrate the effects on male and female rat reproductive performance following Ni NPs treatments during mating, gestation, and lactation. Additionally, the exposure to Ni NPs adversely affected pup survival or development.

It is well known that body weight and organ weight coefficients are sensitive indicators of potentially toxic chemicals in general toxicity studies [44,45]. As described, repeated gavage administration of Ni NPs to rats caused a significant suppression in body weight gain in the male 45 mg/kg group.

At the same time, the decreased ovary weight coefficients and increased testis weight coefficients observed in the Ni NPs and Ni MPs groups are closely related to the treatment of Ni NPs, since correlated histopathological changes such as vascular dilatation and congestion, ovarian lymphocytosis, luteal cells increasing and becoming cavitation, increased eosinophils and inflammatory cell infiltration in female ovary tissues, the shedding of epithelial cells of the raw seminiferous tubule, disordered arrangement of cells in the tube, and appearance of cell apoptosis and death in male testis tissue, were detected on exposure of Ni NPs groups. This interpretation was also supported by the work of Feron in that a changed organ weight coefficient should be considered to be due to an effect of chemicals (*i.e.*, Ni NPs) in cases of growth reduction and organ damage [46]. Although the difference between the groups was not statistically significant in female rats, an increase in lung weight coefficient observed in the male 15 and 45 mg/kg groups was considered to be an adverse effect of the Ni NPs treatment test. This finding suggests that the lung was one of the major targets of Ni NPs in rats.

It is well noted that individual susceptibility to chemical toxicity may be influenced by gender, and differences in physical constitution and physiology may also play a major role in determining gender-specific response and toxicity [47,48]. In the present study, the Ni NPs-related adverse effects on body weight and organ weight coefficients were slightly greater in males than in females perhaps due to lower body fat. Although the exact cause of the gender difference is unknown, the present detailed data on toxicokinetics and metabolism of the test chemicals can provide good information in determining the gender-specific toxicity of Ni NPs.

**Figure 5:**
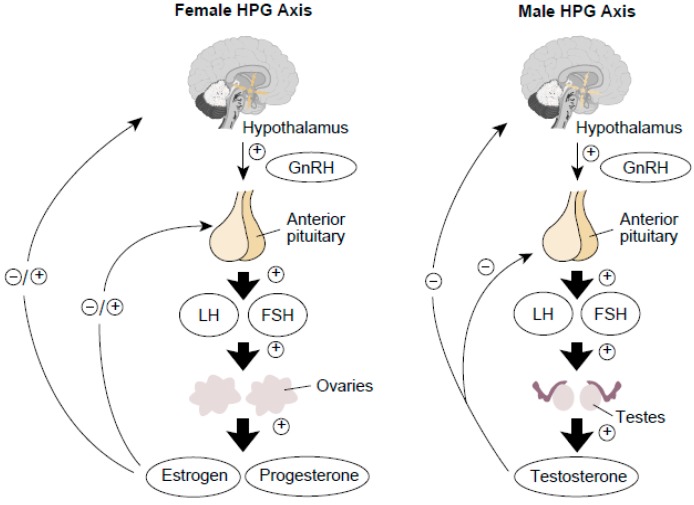
Schematic representation of the hypothalamic–pituitary–gonadal (HPG) axes.

The hypothalamic–pituitary–gonadal (HPG) axis is the hormone system whereby the hypothalamus secretes so-called releasing hormones, which are transported via the blood to the pituitary gland. There, the releasing hormones induce the production and secretion of gonadotropins (*i.e.*, LH and FSH), which in turn are transported by the blood to the gonads (*i.e*., the ovaries and testes). Generally speaking, in females, LH and FSH stimulate the ovarian follicle that contains the maturing egg to produce estradiol. After ovulation has occurred, LH also promotes production of progesterone and E_2_ by the corpus luteum. Both hormones participate in a negative feedback mechanism through most of the menstrual cycle, suppressing GnRH release from the hypothalamus and LH release from the pituitary [49,50]. The mechanism also applies to rat estrous cycles. In males, LH stimulates certain cells in the testes (e.g., Leydig cells) to release T. FSH and T are key regulators of another set of testicular cells (e.g., Sertoli cells), which support and nourish the sperm cells during their maturation. The HPG axis in the male is regulated through a variety of factors [51,52]. For example, T is part of a negative feedback mechanism that inhibits GnRH release by the hypothalamus and LH release by the pituitary. (See Figure 5). Exogenous chemicals can interfere with the normal functioning of the HPG axis, resulting in reduced fertility or even infertility in both females and males. Here we describe the effects of Ni NPs on aspects of serum sex hormone levels (*i.e.*, FSH, LH, E_2_ or T) in female and male rats. The results of the current study demonstrate Ni NPs increased the level of serum FSH and LH, and decreased E_2_ associated with significant and dose-dependent in females. Our results indicate the effects of Ni NPs on the female rat ovarian reserve. It is probably an indication of the decreased level of serum E_2_ and ovarian hormone secretion following ovarian damage with Ni NPs, which increased the level of serum FSH and LH by negative feedback. Meanwhile, the male rat serum FSH, LH and T content analysis showed the levels of FSH and T were decreased significantly by Ni NPs treatment. The results suggest that the decreased level of T, which resulting from testicular damage, affected testicular spermatogenesis. Testicular damage was exacerbated by reduced FSH. From the T value trends, T values reflect the extent of spermatogenic cell damage, and spermatogenesis. The effect of Ni NPs on testicular function in male rats was severe with lowered levels of T. The effects of the Ni MPs on serum sex homone levels are similar to the effects of Ni NPs, but to a lower extent. The change of hormone reproductive levels indicates the abnormal reproductive axis function, which correlated male and female infertility [53]. The current results showed Ni NPs had reproductive toxicity by affecting hormone levels between male and female rats.

Sperm motility can indirectly reflect its fertilizability. There are correlations between fertility rate and motility parameters *in vitro* fertilizing capacity of rat spermatozoa [54]. CASA provides the means for an objective classification of sperm motility. Using digital images of each sperm track, CASA machines are able to analyze, by processing algorithms, the motion properties of spermatozoa. The commonly reported CASA parameters include VCL, VAP, VSL, BCF, ALH, LIN, STR and ELON. These CASA parameters have been modelled and refined mathematically to describe best the motion parameters of each spermatozoon as it travels through a microscopic field [55,56]. The study showed that VCL, LIN and BCF were the most sensitive indicators of male reproductive toxicity, while STR and ELON appeared less sensitive. In this study, it was found that Ni NPs exerted adverse effects on the sperm motility in a dose-dependent manner. The values of VCL and LIN were progressively reduced with increasing exposure dose of Ni NPs. Furthermore, we observed an increase of BCF due to Ni NPs treatment. The data were consistent with our study of histopathology. BCF, along with LIN and VCL, are indicative parameters of spermatic vigor. Observed changes for these parameters in capacitating conditions *in vitro* could be related to sperm hyperactivation that occurs *in vivo* and favors the penetration of oocyte. This may be attributed to diminished fertility of Ni NPs in male. In this study, Ni NPs and Ni MPs were found to exert similar effects on sperm motility, which may be to do with their similar molecular structures. Our results are consistent with the conclusions of Sleiman’s research about Ag NPs which showed Ag NPs had adverse effects on reproductive functions by affecting sperm quality and quantity, and hormone levels *etc.*

In the F0 generation, live birth rate was unaffected by Ni NPs treatment, and mating success rate and pregnancy rate were decreased compared to the control group, but were not significantly different. Additionally, grossly malformed pups were not observed in any groups. However, in the F1 generation, birth survival rate and feeding survival rate were decreased significantly. Ni NPs and Ni MPs caused the loss of neonatal rat weight gain on some days compared with the control group. The possible reason is that the important function of the genes of rat mammary development and lactation was affected by Ni particles resulting in the lower lactation capacity. So the neonatal rats cannot get enough nutrition for their growth and development.

## 4. Experimental Section

### 4.1. Materials, Preparation and Characterization 

#### 4.1.1. Materials of Ni NPs and Ni MPs

Ni NPs, average size 90 nm (Product Code: FNiN-80; Black Powder, Purity: 99%, Surface area ≥ 8 m^2^/g, Bulk density: 0.06–0.8 g/cm^3^) were purchased from Nano Science and Technology Co., Ltd., Kunshan-miyou, Kunshan, China. Ni MPs, average size of 3μm (Product Code: ST-M-008-2; Gray-black powder, Purity: 99.0%, Surface area ≥ 3 m^2^/g) were purchased from Material Technology Co., Ltd. of Shanghai, China.

#### 4.1.2. Preparation of Ni NPs and Ni MPs

Stock suspensions of Ni NPs and Ni MPs were prepared in normal saline (10 mg/mL) by sonication for 30 seconds on ice using a sonifier (Misonix Incorporation, New York, NY, USA). The particle suspensions were kept on ice for 30 s and sonicated again for a total of 3 min at a power of 400 W. Before use, Ni NPs and Ni MPs were diluted to the desired concentrations with 0.9% sodium chloride solution. All samples were prepared under sterile conditions.

#### 4.1.3. Characterization of Ni NPs

The physical properties of Ni NPs were characterized by scanning electron microscope (SEM, JEOL Ltd., Tokyo, Japan) and transmission electron microscopy (TEM, JEOL Ltd., Tokyo, Japan). Then the water-dispersibility and agglomeration state of Ni NPs were studied in normal saline by Zetaszier Nano-ZS (Malvern Instruments Ltd., Malvern, UK).

### 4.2. One-Generation Reproductive Toxicity Test

Male and female Sprague-Dawley rats of 80–100 g were purchased from Shanghai Super-B&K laboratory animal Corp. Ltd. (Shanghai, China) and housed under controlled environment (22 ± 2 °C, 12 h light/dark cycle, free access to food and water) in the Experimental Animal Center, Southeast University (Nanjing, China). The animal approval number was SCXK 2008-0016. All the animal experiments were performed in compliance with the local ethics committee. Animal care and use were in accordance with China’s Guidelines for Care and Use of Laboratory Animals (National Research Council, 1996).

This study was conducted in compliance with OECD guideline test 415: One-Generation Reproduction Toxicity Study. The 50 male and 100 female rats were divided into five groups including control group, Ni NPs (90 nm) groups (high dose 45, mid-dose 15 and low dose 5 mg/kg/day, respectively) and Ni MPs (3 μm) group (45 mg/kg/day) in such a way as to equalize group means and standard deviations of body weights. Each group consisted of 10 males and 20 females, as F0 parental rats. Both male and female F0 parental rats were administered by gavage with different doses of Ni NPs, Ni MPs and 0.9% sodium chloride solution (control group) for 10 weeks before the initiation of the mating period. Females continued to receive test samples during gestation and lactation.

At the end of ten weeks of exposure, the F0 rats were mated on the basis of one male to two females, selected randomly within each dose group for a period of 14 days. The observation of a vaginal plug in a vaginal smear was considered evidence of successful mating. Females were examined daily during the mating period. The day that the vaginal plug in a vaginal smear was observed was designated as day 0 of pregnancy. Once the vaginal plug was observed, the female and male were separated and housed individually in polycarbonate cages. A female was re-mated with a male of proven fertility within the same group if mating was not confirmed within two weeks. All rats were allowed to litter naturally (F1 generation), and rear their own offsprings until weaning.

The male rats were killed at the end of the 14-day mating period, while females that delivered were killed on day 22 after parturition. The undelivered females were killed on day 3 after the last expected parturition date. All male and female rats were subjected to a full and detailed gross necropsy. Special attention was paid to the reproductive organs. At necropsy the following organs were obtained and weighed: liver, kidney, lung, ovary, testis and epididymis.

### 4.3. Sex Hormone Level

After exposure, 4–5 mL of blood per rat was collected and stored in tubes. Collected blood samples were centrifuged at 2500 rpm for 10 min at 4 °C, and the serum was collected and frozen at −80 °C for later analysis. Follicle stimulating hormone (FSH), luteinizing hormone (LH), etradiol (E_2_) and testosterone (T) were measured in serum by competitive enzyme-linked immunosorbent assay (ELISA) kit. All samples and standards were run in triplicate.

### 4.4. Sperm Motility

The motility parameters of sperm were analyzed with a computer-assisted sperm analysis (CASA) system (TOX IVOS, Hamilton Thorne Incorporation, Beverly, MA, USA) as follows: A quantity of 10 µL of sperm diluted solution was placed on observation chambers for CASA analysis. For each male rat, four slides were analyzed. The measured motion parameters were curvilinear velocity (VCL; sum of the incremental distances moved in each frame along the sampled path and dividing by the total time for the track), average path velocity (VAP; a derived path based on an average number of points and divided by the time of the track), straight-line velocity (VSL; the straight-line distance between the start and end points of the track divided by the time of the track), beat cross frequency (BCF; turning points of the sperm head), amplitude of lateral head displacement (ALH; average deviation of the sperm head from the smoothed path), linearity (LIN; straight-line distance divided by the sum of the incremental distances along the actual path × 100), straightness (STR; straight-line distance of the smoothed path divided by the distance along the smoothed path × 100) and Elongation (ELON; the elongation ratio of the minor to major axis of each sperm nucleus).

### 4.5. Histological Examination

The uterus and testis were removed quickly when rats were killed and histological examination was performed following protocols described previously [57]. Briefly, tissues were fixed with 4% paraformaldehyde, routinely processed, and embedded in paraffin and 5 micron in thickness. These sections were stained with hematoxylin-eosin (H&E) for microscopic examination. All tissues taken from the control and high dose groups were examined microscopically.

### 4.6. Data Analysis

Data analysis was performed using the Statistical Analysis Software (SAS 9.1) and Microsoft Excel. The significance of differential expression between groups was assessed by *t*-test and one-way ANOVA. The quantitative data were expressed by mean ± SD, qualitative data were expressed in frequencies, and compared with contingency tables using χ^2^ statistics. Occurrence frequencies were characterised with a Fisher’s exact test. *p* < 0.05 was considered as statistically significant.

## 5. Conclusions

In summary, the findings of this study indicate that Ni NPs can be considered as a reproductive toxicant. In addition, the toxicity observed in the reproductive toxicity studies to both the female rats as well as the male were very similar, in both the severity of the effects and the concentrations at which those effects occurred. Compared with Ni MPs, the toxicity of Ni NPs was more severe in reproductive toxicity studies due to the change of particle size and surface area *etc.* The results of this study will be helpful to further study the long-term effects induced by Ni NPs and the scientific basis for setting standards for safety evaluation for metallic nickel nanoparticles. Further research is needed to elucidate exposure in human populations and mechanism of actions.
